# COVID-19 in Iran: A model for Crisis Management and Current Experience

**DOI:** 10.22037/ijpr.2020.113365.14255

**Published:** 2020

**Authors:** Hamidreza Jamaati, Farzaneh Dastan, Shirin Esmaeili dolabi, Mohammad Varahram, Seyed MohammadReza Hashemian, Shamsi Nasiri Rayeini, Behrooz Farzanegan, Fatemeh Monjazebi

**Affiliations:** a *Chronic Respiratory Diseases Research Center, National Research Institute of Tuberculosisand Lung Diseases (NRITLD), Shahid Beheshti University of Medical Sciences, Tehran, Iran. *; b *Department of Clinical Pharmacy, School of Pharmacy, Shahid Beheshti University of MedicalSciences, Tehran, Iran. *; c *Mycobacteriology Research Center, National Research Institute of Tuberculosis and Lung Diseases (NRITLD), Masih Daneshvari Hospital, Shahid Beheshti University of Medical Sciences, Tehran, Iran. *; d *Tracheal Diseases Research Center, National Research Institute of Tuberculosis and Lung Diseases (NRITLD), Masih Daneshvari Hospital, Shahid Beheshti University of Medical Sciences, Tehran, Iran. *; e *Department of Medical-Surgical Nursing, School of Nursing & Midwifery, Shahid Beheshti University of Medical Sciences, Tehran, Iran.*

**Keywords:** COVID-19, Crisis, Management, Pharmaceutical care, Healthcare, Nursing care

## Abstract

In February 2020, the first sample test was confirmed as positive for corona virus in Masih Daneshvari Hospital that is the reference center in Iran for all pulmonary and respiratory diseases. The decisions made in a hospital or organization to manage a crisis is very vital. Success in managing any crisis requires a scientific and scholarly attitude. This paper was distilled from experiences gained in Masih Daneshvari Hospital in Tehran, capital of Iran, in March 2020 at the stubborn time of coping and managing corona virus crisis. This study was conducted using participatory action research, a methodology which identifies problems in practice, and finds methods to solve them. This Action research involves five stages: statement of the problem, planning, data interpretation and analysis, action, and evaluation of the research process during performing the study. The whole hospital was equipped for corona virus patients in 10 phases during one week and 250 active beds were equipped for these patients. Three models, namely, “corona virus crisis management model”, “Pharmaceutical care management in coronavirus crisis model” and “nursing in coronavirus crisis model” were planned and implemented. During one month of implementing these three models, the supervision team monitored the accurate implementation of instructions and resolving or revising the possible deficiencies and faults. The Masih Daneshvari crisis management model in coronavirus, can be a useful and applicable model in other corona virus centers.

## Introduction

In late December 2019, a group of patients with pneumonia with idiosyncratic etiology were reported to a local healthcare center. The cause of this idiosyncratic pneumonia was a novel corona virus (COVID-19) (1). In February 2020, a total of 33 738 cases and 811 deaths have been reported in China (2). To cope with this disease and the related epidemic, the local government of China devoted several hospitals to these patients as reference hospitals. The contagious nature of the disease demanded another coping strategy presenting a big challenge to ICUs, physicians, and nurses. About %15-20 of the patients suspected of and confirmed with corona virus COVID-19 developed hypoxia since the second week of affliction that finally needed assisted ventilation methods such as high-flow nasal cannula and invasive or noninvasive mechanical ventilation. In addition to severe hypoxemia and reduced tissue perfusion, the patients suffered from complications like shock, acute kidney injury, gastrointestinal bleeding, and rhabdomyolysis. No effective specialized antiviral drug has been developed yet to fight corona virus. Thus, the management of these patients is based on basic and supportive care. Since there is no definitive cure for this disorder, giving care to these patients that are often exposed to acute respiratory distress syndrome, imposes much workload on physicians and nurses, especially in respiratory care wards. The more patients are affected with acute respiratory distress syndrome induced by this condition, the greater the need for ICU, equipment, and trained and experienced staff will be (3). 

The decisions made in a hospital or organization to manage a crisis is very vital. These decisions ought to be made rapidly without wasting any time. Highly experienced individuals should manage the crisis, and they should know that making any wrong decision may result in great negative consequences. Success in managing any crisis requires a scientific and scholarly attitude. Crisis management is considered as a long, complex and technical process. This paper was distilled from experiences gained in Masih Daneshvari Hospital in Tehran, capital of Iran, in March 2020 at the stubborn time of coping and managing corona virus crisis. 

## Experimental

This study was conducted using participatory action research, a methodology which identifies problems in practice, and finds methods to solve them. Action research is applied as an effective research method, leading to facilitation of changes and promotion of services in industry and education, and recently, in healthcare. This method improves the clinical care, team work, communication, and clinical management (4). Action research involves five stages: statement of the problem, planning, data interpretation and analysis, action, and evaluation of the research process during performing the study (5). The goal of action research is to increase knowledge and empower individuals that are exactly involved in a phenomenon (6). Having defined the problem as a “viral respiratory disease crisis”, a plan was offered to cope with it. After data analysis and interpretation, three models, namely, “corona virus crisis management model”, “Pharmaceutical care management in coronavirus crisis model” and “nursing in corona virus crisis model” were planned and implemented. During one month of implementing these three models, the supervision team monitored the accurate implementation of instructions and resolving or revising the possible deficiencies and faults. 

## Results

In March 2020, the first sample test was confirmed as positive for corona virus in Masih Daneshvari Hospital that is the reference center in Iran for all pulmonary and respiratory diseases. This hospital had experienced coping with influenza in 2009. The crisis management team was formed by the head and vice-president of the hospital and major decisions for managing corona virus crisis were made. Given that corona virus was a viral contagious respiratory disease, crisis coping was divided into ten phases to manage it correctly, avoid confusion and mess, and prevent fear and anxiety in the staff. To do so, Ward 3 of the hospital was selected as the pilot ward. 

All patients in this ward were transmitted to other wards or hospitals in less than 24 h. This ward enjoyed experienced nurses, knowledgeable head nurses, and 30 beds. The staff received personal protective equipment and all trainings related to personal protection were provided again. Some information on corona virus was given to the staffs. The benefit of this type of planning was that it clarified the amount of equipment and supplies required for managing the crisis, the number of hospitalized patients, the nursing approach and performance, services, nurses’ and staff’s fears and anxiety, and pharmaceutical management.

 Having announced Masih Daneshvari Hospital as the reference hospital in Tehran, 30 corona virus patients were hospitalized there in less than 24 h. Hence, the management team decided to implement the second phase wherein wards 4 and 6 were equipped for hospitalization of these patients. Besides, the knowledge and experiences of nurses in Ward 3 were given to these nurses. Subsequently, the ICU for TB patients was converted to corona virus ICU. Then, the CCU patients were transferred to other hospitals and the CCU was ready to admit corona virus patients. At the completion of the capacity of these wards, the surgical ward 1 and 2 and surgical ICU ward 1 and 2 were also ready to admit corona virus patients. 

For the hospital to concentrate on corona virus crisis and to avoid the probability of spread of the disease to patients undergoing surgery, all operations were cancelled. All out-patient clinics were off and all hospital activities focused on care-giving and hospitalization of corona virus patients. Considering the mean presentation of 300-400 cases per day to the hospital Emergency Room and triage, three triage wards were activated to avoid patient pile-ups.

**Figure 1:**
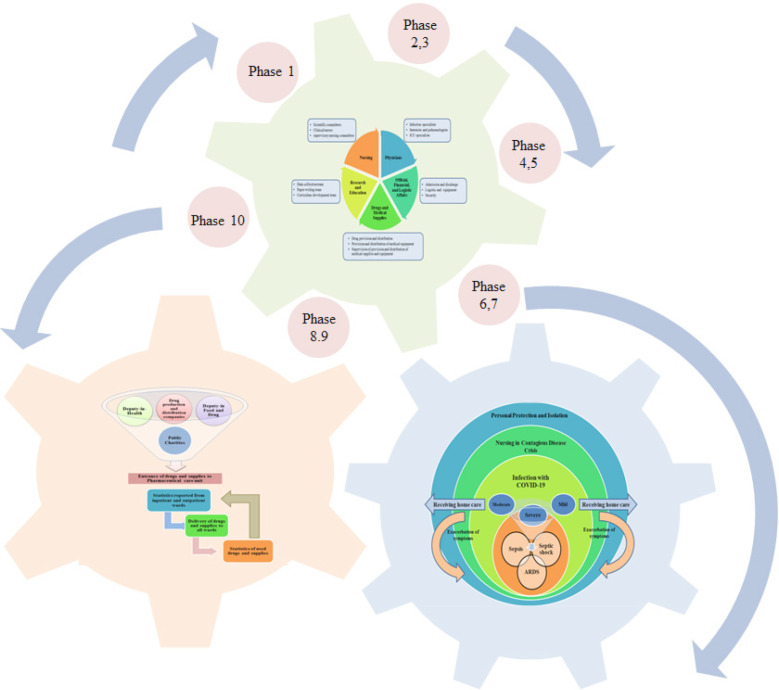
Crisis Management Model

**Figure 2:**
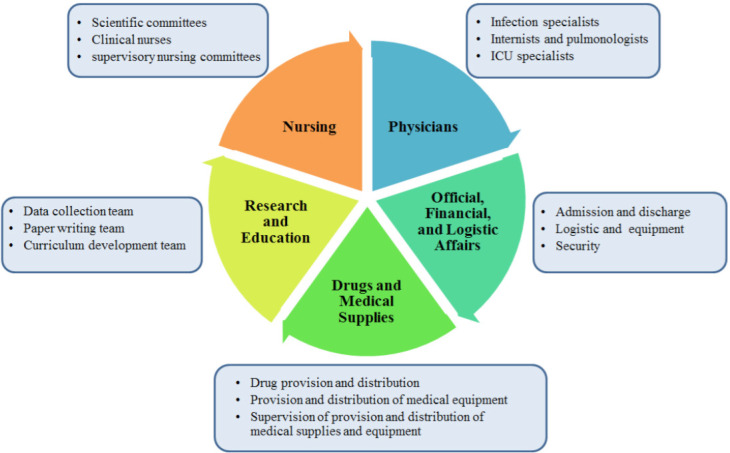
Management Model in Coronavirus Crisis

The whole hospital was equipped for corona virus patients in 10 phases during one week (Figure1). Also, 250 active beds were equipped for these patients. The crisis management team divided the management in 5 sections among the managers. In so doing, the daily planning, patient visits, and on-call shift was devoted to a specialist; all 400 nursing staff worked under the supervision of hospital matron; the official and financial affairs and hospital equipment and supplies were supervised by hospital management; the pharmacy, protective and therapeutic equipment were supervised by the hospital pharmacist; hospital research ward was managed by vice-chancellor in research who collected data, wrote paper drafts, and reported patient management. This submission of responsibilities and duties removed the bureaucratic need for holding time-consuming sessions and meetings and the mangers of any ward could make proper decisions at any time (Figure2). 

**Figure 3.**
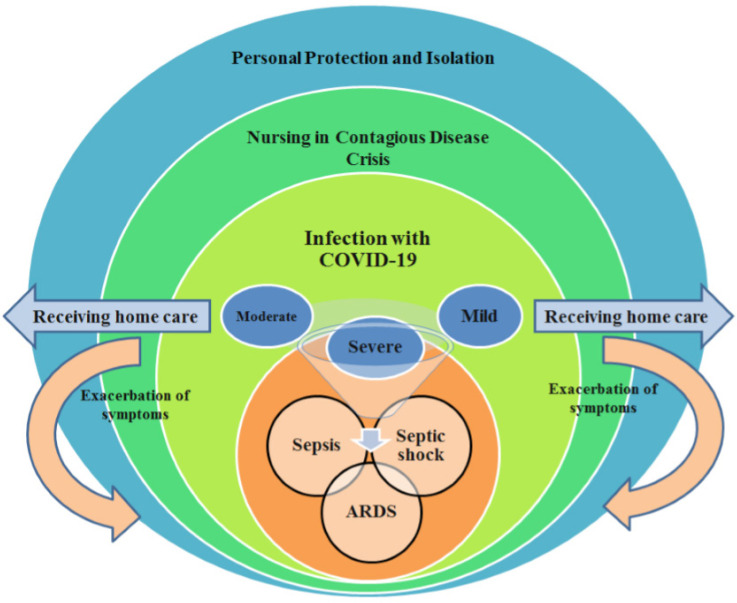
Nursing Care Model in Coronavirus Crisis

The Nursing Committee developed a model of giving care to corona patients after initial visits of nursing wards, interviewing physicians and nurses, and holding numerous sessions for planning care-giving of these patients (Figure3). Planning by Scientific Committee of Nursing on this basis determined the responsibility of developing educational, managerial, and supervisory materials. 

The importance of transmission of the disease in a study in China shows that 41% of cases of coronavirus occur in hospitals. This includes patients currently hospitalized and the health care team, which includes physicians and nurses, radiologists and all those who are in direct contact with patients. Another issue is the transmission from one patient to another (7). Given the surveys conducted by Nursing Committee and considering the statistics reported on the rate of nurses’ affliction with corona in the hospital under study, that increased progressively, full instructions were given again to nurses in all wards regarding method of wearing protective gowns (spacemen gown), hat (headgear), gloves, goggles, face shields, and masks (8). 

It was also decided that all nurses go to breakfast and lunch with the same clothes they wore at the onset of their work shift and avoid pile-ups at the meal times. Food and breakfast were given to nurses as packaged meals/snacks and all nursing staff was required to wear their face shields and goggles from the beginning of their work and avoid any contact of hands with their eyes. All nurses were obliged to wear double-gloves during working with patients though they could wear single-gloves when writing reports. The use of latex gloves was preferable in all treatment wards (8). According to the study in China, nurses’ shoes and socks/stockings were the most contaminated parts; hence, it was advised that nurses wear high-heel shoes that are highly simple and can be sterilized with alcohol. Given the viral disease crisis, the managerial structure and nursing shift timings ought to be modified to protect the staff against fatigue and disease. Nurses’ work plan was determined daily. Nurses’ work shifts were designed as 12-h shifts without the need for sleeping (9).

Because the sleeping rooms are not separate in the hospital pavilion, so, it is highly possible that previous nurses might have contaminated the beds. Also, taking off clothes might infect nurses. Considering that personal protective equipment is made of fibers and materials that induce sensitivity in some individuals and given that they cost much money, planning work shifts as 12-h periods is the best method of preventing increased use and chances of sensitivity in nurses (10). Dormitories outside of hospital were provided to be used by nurses if necessary. The supervisors had to receive patient reports on the phone or by pager or messenger (10,11). They should avoid attending patients’ bedside one by one. Nurses working in other hospitals could help voluntarily those employed in the hospital under study (12). In this way, nurses’ workload was diminished and the odds of their fatigue and affliction were decreased. Some studies have demonstrated that nurses are exposed to various physical and mental problems during corona crisis. High workload due to simultaneous hospitalization of great numbers of patients with a disease with no known cure, concern with personal protective equipment, chances of affliction with the disease, odds of family and children’s affliction with the disease and the possibility of their being carriers along with other problems and also the social problems associated with a crisis in the community all expose nurses to psychological damages and exhaustion, finally culminating in reduced quality of care study (12). Therefore, Committee of Nursing at Masih Daneshvari Hospital attended hospitalization wards twice a week and provided nursing education and training required for managing stress and fear in nurses. Besides, infection control supervisor ensured all nurses of securing personal protective equipment. Nurses who used Immunosuppressant, were diabetic or pregnant, or suffered from immunodeficiency (10 participants) went to obligatory vacation (13). Any crowd of visitors or attendants should be prevented (10). All patient information regarding their condition and course of treatment should be given to the patient’s representative or through the virtual messengers. Next, the supervision team assessed again twice a week the nurses’ performance. Two nurses were affected with corona virus at the onset and 8 more nurses at the end of 3 weeks. Again, the supervision committee investigated the causes of increased number of afflicted nurses. An important point pertained to wards without a suitable ventilation system or where nurses did not observe the personal protection. Thus, nurses were provided again with personal protection instructions during the work rounds of supervision team and scientific committee. To ward off the staff’s moving back and forth, food items were given to all staff as packaged meals. Ten nurses left work that returned to work after two weeks. At the end of the fourth week, 27 nursing and official staff were affected with corona virus. Telegram channel was installed for the affected staff. They were all asked to announce their symptoms and problems in this group and they were contacted daily by the phone. To better familiarize the nurses with signs and symptoms of the disease, the scientific committee of nursing decided to teach all signs, symptoms and action of corona virus to them through voice messages. These instructions included viral infection with corona virus, signs and symptoms, and patient management in various conditions. To follow up patients after hospital discharge, a 4-member team of nurses was formed that received the required education on drugs and their complications and self-care in a short time. They provided some guidance for patients on the phone regarding isolation/quarantine method, personal self-care, disease complications, and the administered drugs. The triage physicians and nurses explored regularly the latest guidelines related to patient hospitalization and triage. All patients in this hospital were visited daily by four specialists in infectious diseases. Furthermore, all ICU patients were visited daily by four ICU subspecialists. These physicians followed the same protocol for decision-makings on intubation, mechanical ventilation, weaning the patient from ventilator, noninvasive mechanical ventilation, and patient care. The heads of each ward also visited the patients daily. 

**Figure 4:**
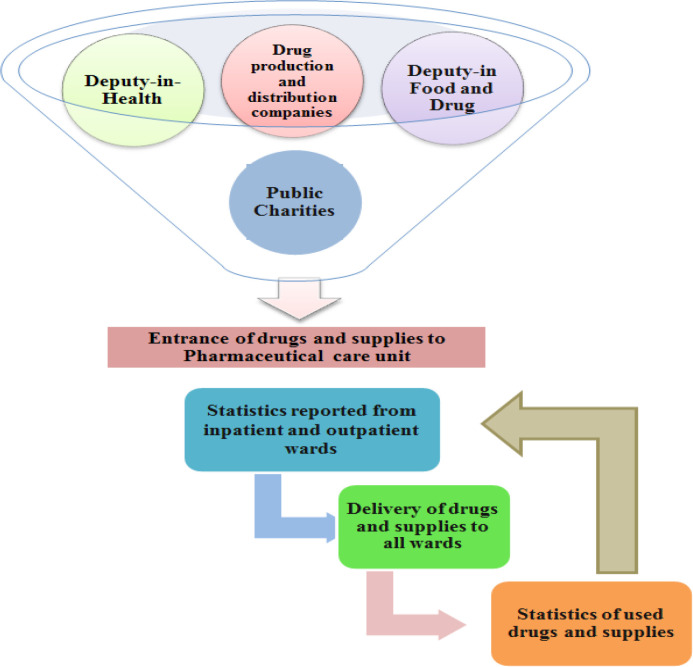
Pharmaceutical Care Management Model in Coronavirus Crisis

The manager of medicinal care ward estimated a list of medical equipment and supplies and the required medicines like antiviral, antibiotic, serums, electrolytes, etc. along with personal protective equipment such as isolation gown, mask, hat, shoe cover, face shield, and goggles and also hospital requirements like radiology appliances, ECMO set, CRRT set etc. for 8 months and sent it to Deputy-in-Food and Drug (14). At the end of the first week, the number of hospital presentations, number of hospitalized patients, number of physicians and nurses and those that needed personal protective equipment, and number of inpatients and outpatients that needed drugs were listed again more accurately and sent to the Deputy-in-Food and Drug. Some antivirals were obtained from deputy-in-food and drug on a rationing basis, some from deputy-in-health, and some from drug dispensers. Moreover, some drugs were bestowed to the hospital by charity organizations. To distribute drugs, the daily required antiviral dosage of each patient was determined first on the basis of the national protocol. Then, the required drugs were received from medicinal care unit on the basis of the complete list of patients and submitted to the hospital pharmacy, which were subsequently delivered to inpatient wards. Finally, after 24 h, the statistics of drugs and supplies were again determined on the basis of patient and staff statistics and given to medicinal care unit (Figure4). In this way, sure was made that the drugs and supplied were used in the correct path. 

## Discussion

From 20 of February to 23 of April, 1745 patients with COVID-19 were hospitalized in Masih Daneshvari Hospital. PCR test showed that 1200 of them were definitely positive for corona virus. Of these, 196 passed away. The mean hospital presentation was 300 patients per day of whom 15 were hospitalized per day. These patients were hospitalized with symptomatic pulmonary CT scan and acute respiratory distress syndrome. The reduction in hospital presentations after the third week was due to the increased number of hospitals that admitted corona patients. The high mortality rate (%11/2) in this hospital was due to its being the reference hospital for respiratory disorders in Iran as highly experienced practitioners worked there. Hence, many of the admitted patients were critically ill patients and were admitted here with acute respiratory distress syndrome or even shock from other hospitals. 

The importance of contagion of the disease in the study in China shows that %41 of infection with corona virus occurs in the hospital. This includes patients already hospitalized in wards and also the healthcare team, i.e., physicians, nurses, radiology technicians and all individuals that are directly in contact with patients. Another noticeable issue is transfer of disease from one patient to another (7). In Masih Daneshvari Hospital 143 of nurses, doctors and staffs were affected with corona virus. Three nurses that left their work returned to work after a while. Making models and showing staffs the way of doing their duty very simple and by graphs, in a crisis which all staffs are stress full, help them to be more effective and cop with their fears.

The ‘order’ in a viral disease crisis ought to be ‘minimal contact and greatest services’. To do so, all staffs should plan to meet all of the patient’s therapeutic needs and interventions concurrently when she/he is at patient’s bedside; for example, she must meet the patient’s needs for food, drug, vital signs, and serum change and lab test at one time (8).

Our findings showed that phasing the hospital for converting it to corona hospital improved the managerial performance and control of the condition and helped in enhancing patient organization and hospitalization. Phasing increased opportunities of examining protective needs of the staff and prevented lack of confidence in the healthcare system or creation of mess in the hospital. The use of crisis management model based on coordination, cause to promotion of hospital preparation (15). 

Janius stated that in a crisis management model, coordination among state health departments, hospital managements, concession companies and utility providers must be developed (16). In our crisis management model all of them were coordinate. There are several models for crisis management. The difference between our crisis management model and other models was that it was presented and implemented simultaneously in the crisis.

The novelty aspects of our model were that Masih Daneshvari was the first hospital in Iran which hospitalized patients with COVID-19 and had the highest number of outpatients and inpatients. By presenting this model and implementing it, the hospital was able to overcome the problems caused by the crisis and act well in treating patients. Having a crisis management model for Hospitals is essential for being responsive against internal and external risks.

## Conclusion

The Masih Daneshvari Crisis Management Model can be a useful and applicable model in other corona virus centers. 
